# Prognostic Significance of *CLDN1*, *INHBA*, and *CXCL12* in Colon Adenocarcinoma: A Multi-Omics and Single-Cell Approach

**DOI:** 10.3390/biomedicines13051035

**Published:** 2025-04-24

**Authors:** Jaehwan Cheon, Sang Hyun Kim, Jaehyung Park, Tae Hoon Kim

**Affiliations:** 1 Department of Otorhinolaryngology-Head & Neck Surgery, Korea University College of Medicine, Anam-ro 145, Seongbuk-gu, Seoul 02841, Republic of Korea; 2 Department of Biomedical Science, Korea University College of Medicine, Anam-ro 145, Seongbuk-gu, Seoul 02841, Republic of Korea; 3 Department of Internal Medicine, Korea University College of Medicine, Anam-ro 145, Seongbuk-gu, Seoul 02841, Republic of Korea; 4 Mucosal Immunology Institute, Korea University College of Medicine, Anam-ro 145, Seongbuk-gu, Seoul 02841, Republic of Korea

**Keywords:** colon adenocarcinoma, bioinformatics, biomarker, *CLDN1*, *INHBA*, *CXCL12*

## Abstract

**Background/Objectives**: Colon adenocarcinoma (COAD), the most prevalent form of colorectal cancer, remains a leading cause of cancer-related mortality. Advances in various treatments for COAD have significantly improved treatment outcomes. However, therapeutic limitations persist, highlighting the need for personalized strategies driven by novel biomarkers. The aim was to identify key hub genes that could be potential biomarkers of COAD using comprehensive bioinformatic analyses. **Methods**: Differentially expressed genes (DEGs) and co-DEGs were identified from COAD gene expression datasets. Functional enrichment analyses, including Gene Ontology (GO) and Kyoto Encyclopedia of Genes and Genomes (KEGG) pathway analysis, were performed. Hub genes were extracted from protein–protein interaction (PPI) networks and validated epigenetically using microRNA (miRNA) and DNA methylation datasets. Their expression patterns were further examined via single-cell RNA sequencing (scRNA-seq) and immune cell infiltration analysis. Prognostic relevance was assessed based on tumor metastasis and survival outcomes. **Results**: Gene expression profiling identified 118 co-DEGs, with GO and KEGG pathway analyses revealing significant pathway enrichment. PPI network analysis pinpointed 27 key co-DEGs. Epigenetic profiling indicated that both miRNA interference and DNA methylation regulate *CLDN1*, *INHBA*, and *CXCL12* expression levels. scRNA-seq analysis showed elevated *CLDN1* expression in epithelial cells and *INHBA* in myeloid cells, and reduced *CXCL12* expression in stromal cells. Prognostic analysis further demonstrated that *CLDN1* and *INHBA* are significantly associated with poor COAD outcomes. **Conclusions**: We identified some potential prognostic biomarkers for patients with COAD. Further experimental validation is required to translate these findings into precision medicine for COAD.

## 1. Introduction

Colorectal cancer (CRC) is the third most prevalent cancer, impacting approximately 1 in every 23 men and 1 in every 25 women [1]. CRC represents 8% of all cancer-related fatalities, ranking it as the second leading cause of cancer mortality globally [2]. Significant improvements in colon cancer treatment have emerged over the years, resulting in better survival rates and quality of life for patients. The notable improvement of colon cancer treatment can be attributed to advancements in a wide array of medical treatments, including laparoscopic surgery, radiotherapy, neoadjuvant and palliative chemotherapies, and targeted therapies [3]. This progress is further supported by an enhanced understanding of the epidemiology, pathology, and molecular mechanisms associated with CRC [4]. However, there are still patients with CRC who face limitations in treatment, prompting new attempts to improve cure rates [5]. It is widely recognized that patients with CRC often exhibit varying treatment responses and prognoses, even when their tumors are histologically identical [6,7]. Consequently, personalized treatments guided by novel biomarkers are expected to yield substantial clinical efficacy and hold significant public health value [8,9].

The tumor micro-environment (TME) plays a crucial role in the progression and treatment of cancer [10,11]. TME comprises cancer cells, immune cells, stromal cells, extracellular matrix components, and signaling molecules that interact dynamically to influence tumor growth, invasion, and metastasis [11]. Targeting the TME has emerged as a promising strategy to enhance cancer treatment efficacy [12,13]. MicroRNAs (miRNAs) and DNA methylation play critical roles in the regulation of the TME [14,15]. miRNAs are short non-coding RNAs of approximately 18–25 nucleotides in length [14]. Extensive research has demonstrated the aberrant expression of miRNAs in CRC [10]. MiRNAs can function as either tumor suppressors or oncogenes in tumor tissues in CRC. In CRC, miRNAs play a crucial role in regulating and suppressing various signaling pathways, offering significant promise for diagnosis, prognosis, and personalized targeted treatment [16]. DNA methylation is an epigenetic mechanism that often leads to gene silencing when occurring in promoter regions of genes [15]. In CRC, certain crucial tumor suppressor genes can be silenced through hyper-methylation, and oncogenes can be activated by hypomethylation processes [15].

As bioinformatics techniques become more advanced and sophisticated, the discovery and characterization of differentially expressed genes (DEGs) as hub genes in diseases like colon adenocarcinoma (COAD)—which constitutes around 95% of CRC—are accelerating. Although several previous studies have explored biomarkers in COAD using transcriptomic or epigenomic data independently, few have attempted to comprehensively integrate multi-omics data—including gene expression, miRNA regulation, DNA methylation, and single-cell RNA sequencing (scRNA-seq)—to identify robust and clinically relevant hub genes. This study is among the first to combine these diverse analytical layers with immune infiltration and prognostic analyses to systematically investigate the TME and molecular mechanisms of COAD.

In this study, we have focused on hub genes that interact with miRNAs and methylation changes, identifying novel biomarkers that are not only diagnostic but also predictive of response to immunotherapies, ultimately advancing personalized medicine in COAD oncology. Using five GSE (gene expression omnibus series) datasets composed of colon tissue, we identified hub genes of COAD. We then utilized the COAD miRNA dataset to identify significant miRNAs and correlated them with the previously identified hub genes. We also analyzed the methylation status of hub genes through bioinformatics, classifying genes with significant methylation changes. We conducted further immunologic analysis on the selected hub genes to confirm their potential as valuable biomarkers for future use.

## 2. Materials and Methods

### 2.1. Microarray Data

The Gene Expression Omnibus (GEO), hosted by the National Center for Biotechnology Information (NCBI), is an openly accessible repository found at https://www.ncbi.nlm.nih.gov/geo/ (accessed on 15 January 2024). We retrieved five COAD gene expression datasets from GEO using keywords such as ‘Colorectal cancer’ and ‘COAD’ and analyzed them via GEO2R (https://www.ncbi.nlm.nih.gov/geo/info/geo2r.html, accessed on 15 January 2024) [17]. These datasets include GSE37364 (10 COAD tissues and 10 normal colonic mucosa), GSE41657 (25 COAD tissues and 12 normal epithelium or colorectal mucosa), GSE44076 (98 COAD tissues and 50 normal mucosa), GSE110224 (17 COAD tissues and 17 normal tissues), and GSE115261 (10 COAD tissues and 10 normal tissues). Since datasets used were obtained from publicly available repository, ethical review and approval were waived for this study.

### 2.2. Identification of Differentially Expressed Genes (DEGs) and Co-DEGs

Using GEO2R, we analyzed DEGs between COAD and normal tissues across all five datasets. We identified DEGs within each dataset (GSE37364, GSE41657, GSE44076, GSE110224, and GSE115261) based on an adjusted *p*-value < 0.05, and a log_2_ fold change (log_2_FC) threshold of |log_2_FC| > 1.5. After detecting DEGs in each dataset, a cross-analysis of these five datasets was performed using a Venn diagram to detect co-DEGs.

### 2.3. Gene Ontology (GO) and Kyoto Encyclopedia of Genes and Genomes (KEGG) Pathway Analysis of Up- and Down-Regulated Co-DEGs

GO and KEGG enrichment analyses were conducted using the Database for Annotation, Visualization, and Integrated Discovery (DAVID) (https://david.ncifcrf.gov, accessed on 5 February 2024) (v7.0) [18].

### 2.4. Protein–Protein Interaction (PPI) Network Buildup on Up- and Down-Regulated DEGs for Hub Genes Detection

PPI networks were constructed using STRING (v12.0) (https://string-db.org/, accessed on 5 February 2024) [19].

### 2.5. Analysis of Differentially Expressed miRNAs (DEMs) Related to the Hub Gene Expression

We detected DEMs using the criteria of an adjusted *p*-value < 0.05 and threshold of |log_2_ FC| > 1. These analyses were conducted across three selected non-coding RNA profiling datasets: GSE18392 (116 colon tumors and 29 normal colon samples), GSE35982 (8 colorectal cancer tumors and 8 normal colorectal tissue samples), and GSE41655 (33 COAD tissues and 15 normal colorectal mucosa samples) obtained from GEO2R. Following the identification of up-regulated and down-regulated DEMs and co-DEMs across three datasets, we utilized The University of Alabama at Birmingham CANcer data analysis Portal (UALCAN) database (https://ualcan.path.uab.edu/analysis.html, accessed on 19 February 2024), which operates based on The Cancer Genome Atlas (TCGA) [20] for validating the expression levels of the co-DEMs in COAD and identification of key DEMs. Subsequently, we investigated the target genes of key co-DEMs to assess their influence on hub gene expression in COAD using ENCORI/starBase (v3.0) (https://rnasysu.com/encori/, accessed on 19 February 2024) [21]. As miRNA typically inhibits the transcription of target genes [22], we examined the negative correlation between expression of these key co-DEMs and hub genes.

### 2.6. Analysis of Differentially Methylated Regions (DMRs) of Hub Genes

We identified DMRs of the CpG island, expression-regulatory elements of a gene, such as promoters and enhancers, using the criteria of an adjusted *p*-value < 0.05 and a threshold of |log_2_FC| > 0.015. This analysis was conducted on the GSE42752 dataset, which includes 22 COAD and 41 normal colon genomic DNA samples obtained from GEO2R. As hyper-methylated DMRs of specific genes typically lead to the transcription inhibition of the gene [23], we analyzed the negative correlation between methylation levels of DMRs and expression levels of hub genes. Moreover, we corroborated the level of promoter methylation and expression of hub genes by scrutinizing UALCAN for using TCGA dataset and validation.

### 2.7. Hub Gene Expression Pattern Analysis in the COAD scRNA-Seq Dataset

For exploring the expression patterns based on cell types of some hub genes in COAD, we opted for the GSE178341 dataset, consisting of 62 COAD tumor (total 258,359 cells) and 36 normal colon tissues (total 112,864 cells) using the Single-Cell Portal (SCP) database (https://singlecell.broadinstitute.org/single_cell, accessed on 12 February 2024) [24].

### 2.8. Analysis of Immune Cell Infiltration Level

We utilized the TIMER 2.0 database (http://timer.cistrome.org/, accessed on 12 February 2024) to compute the correlations between the expression levels of hub genes and the infiltration of various immune cell types in COAD (*n* = 458) [25].

### 2.9. Tumor Metastasis Analysis According to Hub Gene Expression

We assessed the influence of specific hub gene expression on tumor metastasis in patients with COAD to investigate its effect on tumor prognosis using the Tumor, Normal, and Metastatic tissues (TNM) plot.com database (377 normal, 1450 tumor, and 99 metastatic samples) (https://tnmplot.com/, accessed on 19 February 2024) [26].

### 2.10. Analysis of Survival Rates According to Hub Gene Expression

We employed the Kaplan–Meier (KM) plot database (https://kmplot.com/, accessed on 19 February 2024) to explore the correlation between the expression levels of specific hub genes and patient survival in individuals with COAD [27]. It was utilized to assess overall survival (OS) in patients with COAD (*n* = 1061) for certain hub genes.

### 2.11. Data Visualization

All volcano plots, heatmap plots, and bar and bubble plots, illustrating the GO and KEGG pathways, were generated using Hiplot (https://hiplot-academic.com/, accessed on 8 February 2024) [28].

**Figure 1:**
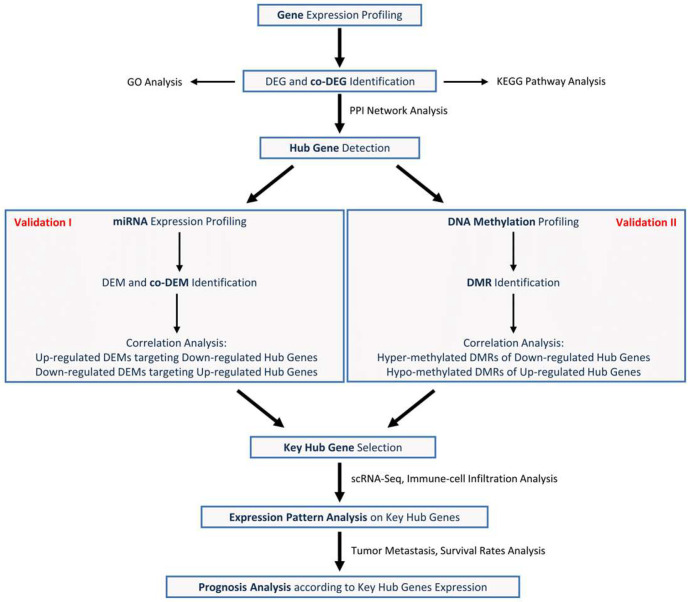
Flowchart used in this study.

Taken together, Figure 1 exhibits the overall research flow used in this study.

## 3. Results

### 3.1. Identification of DEGs and Co-DEGs in Five COAD Gene Datasets

**Figure 2:**
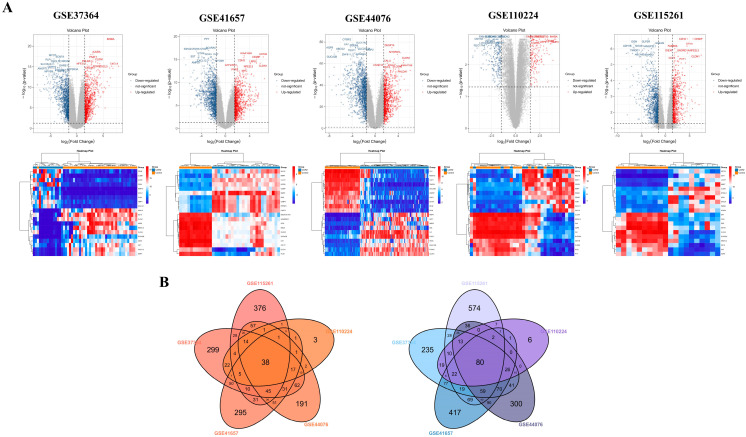
Identification of DEGs and detection of co-DEGs of COAD using five gene expression datasets. Volcano map illustrating differentially expressed gene (DEG) distribution and heatmap showing representative 10 up-regulated and down-regulated DEGs in GSE37364, GSE41657, GSE44076, GSE110224, and GSE115261 datasets (**A**). The red points in the volcano plots indicate up-regulated genes identified with a fold change of ≥1.5 and a corrected *p*-value of <0.05. Conversely, the blue points represent down-regulated genes identified with a fold change ≤ −1.5 and a corrected *p*-value < 0.05. Black points denote genes with no statistically significant difference. Gene expression is depicted in a heatmap using color coding. Red indicates up-regulation, blue denotes down-regulation, and white indicates no significant change. A total of 38 up-regulated and 80 down-regulated co-DEGs are identified through analysis of the cross-linking data from GSE37364, GSE41657, GSE44076, GSE110224, and GSE115261, and visualized using a Venn diagram (**B**).

Using GEO2R, we analyzed five datasets (GSE37364, GSE41657, and GSE44076, GSE110224, GSE115261) and identified a total of 3329 up-regulated and 4782 down-regulated DEGs. Reciprocal volcano maps for each dataset illustrate the distribution of significantly altered genes (Figure 2A). Representative heatmaps showcase 20 DEGs in each dataset (Figure 2A). Notably, cross-analysis revealed 118 co-DEGs (38 up-regulated and 80 down-regulated), visualized in a Venn diagram (Figure 2B).

### 3.2. GO and KEGG Pathway Analysis on Up- and Down-Regulated Co-DEGs

**Figure 3:**
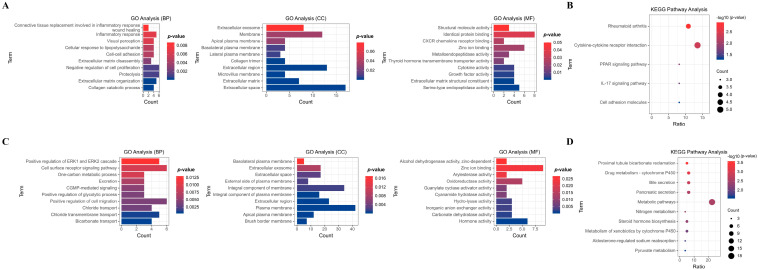
GO and KEGG analyses of co-DEGs in the five datasets. GO analysis categorized the up-regulated and down-regulated co-DEGs into several biological pathways (BPs), cellular components (CCs), and molecular functions (MFs), based on their roles (**A**,**B**). Furthermore, KEGG pathway analysis is used to classify the up-regulated and down-regulated co-DEGs biochemical pathways according to their gene functions (**C**,**D**).

To comprehend the functional implications of co-DEGs, we conducted GO and KEGG pathway analyses for each up- and down-regulated co-DEGs. As illustrated in Figure 3A, up-regulated co-DEGs showed significant enrichment in biological pathways (BPs) related to proteolysis, extracellular matrix (ECM) organization, inflammatory response, and other associated processes. Additionally, they were notably enriched in cellular components (CCs) associated with the extracellular space, extracellular region, membrane, and other components. Moreover, they exhibited significant enrichment in molecular functions (MFs) related to identical protein binding, zinc ion binding, and serine-type endopeptidase activity, among others. KEGG pathway analysis further underscored specific pathways enriched with up-regulated co-DEGs. These included significant enrichment in rheumatoid arthritis, cytokine–cytokine receptor interaction, the interleukin (IL)-17 signaling pathway, and others (Figure 3B).

Conversely, down-regulated co-DEGs demonstrated significant enrichment in biological pathways associated with positive regulation of cell migration, cell surface receptor signaling pathways, positive regulation of extracellular signal-regulated kinase (ERK) 1 and ERK2 cascades, and other related processes. Additionally, they were notably enriched in cellular components associated with the plasma membrane, integral components of the membrane, extracellular regions, and other components. Moreover, they exhibited significant enrichment in molecular functions related to zinc ion binding, hormone activity, oxidoreductase activity, and other functions (Figure 3C). KEGG pathway analysis revealed enrichment in metabolic pathways, steroid hormone biosynthesis, bile secretion, and other pathways (Figure 3D).

### 3.3. PPI Network Construction of Co-DEGs and Detection of Hub Genes

**Figure 4:**
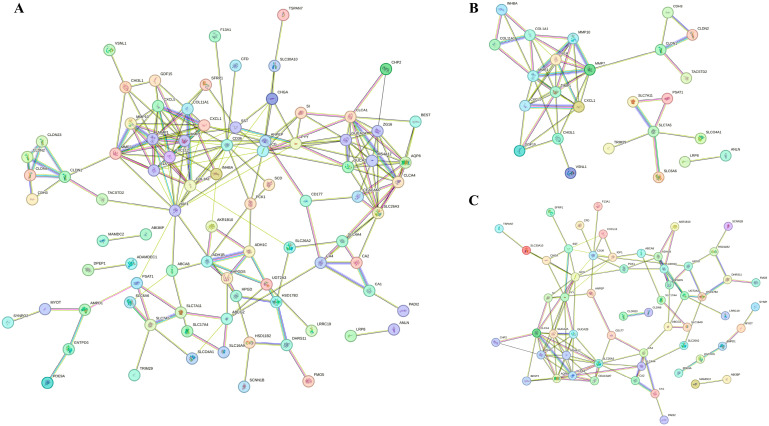
PPI network diagrams of co-DEGs. Each diagram represents a network of all co-DEGs (**A**), up-regulated DEGs (**B**), and down-regulated DEGs (**C**). Each network represents a gene, and each line represents the interaction of proteins. The results within the circle represent protein structure. The color of the line represents evidence of the interaction.

For the identification of key genes potentially influencing the progression of COAD, we scrutinized all 118 co-DEGs using the STRING database to construct PPI networks. Co-DEGs showing connectivity above six were considered hub genes, unveiling numerous promising contenders. Notably, insulin-like growth factor (IGF)1 emerged with the highest connectivity at sixteen, followed by matrix metalloproteinase (MMP)1 at fourteen, and others, including cluster of differentiation (CD)36 (node degree of thirteen), collagen type I alpha 1 chain (COL1A1) (node degree of eleven), C-X-C motif chemokine ligand (CXCL)12 (node degree of eleven), claudin (CLDN)1 (node degree of six), and inhibin β (INHB)A (node degree of six). In total, 27 hub genes were filtered from the 118 co-DEGs (minimum required interaction score = 0.4, *p* < 1.0 × 10^−16^) (Figure 4A). Additionally, PPI networks of up-regulated (minimum required interaction score = 0.4, *p* < 1.0 × 10^−16^) (Figure 4B) and down-regulated co-DEGs (minimum required interaction score = 0.4, *p* < 1.0 × 10^−16^) (Figure 4C) were constructed.

### 3.4. Identification of DEMs Regulating Hub Gene Expression in Three COAD Datasets

**Figure 5:**
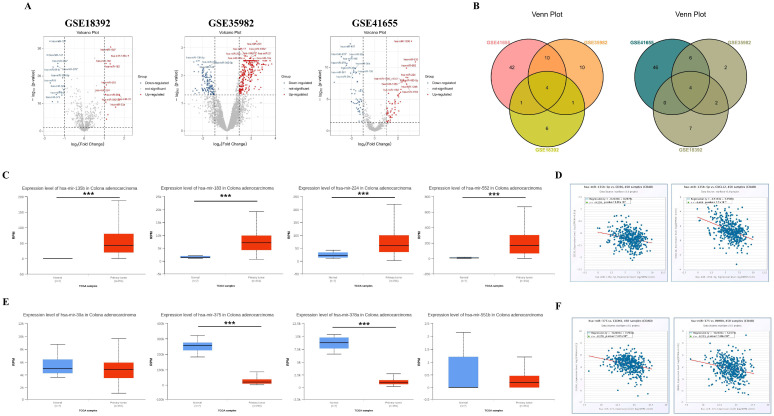
Some hub gene expression levels are regulated by miRNA interference. Volcano maps showing the distribution of differently expressed microRNAs (miRNAs) (DEMs) in GSE18392, GSE35982, and GSE41655 (**A**). In the volcano plots, red points indicate significantly up-regulated DEMs identified with a fold change ≥ 1.0 and a corrected *p*-value of <0.05. On the contrary, blue points represent down-regulated DEMs identified with a fold change ≤−1.0 and a corrected *p*-value of <0.05. The co-DEMs in the three miRNA datasets were separated using a Venn diagram of the up-regulated and down-regulated DEMs, respectively (**B**). All four up-regulated co-DEMs are expressed at a higher level in the TCGA database (**C**), and only hsa-135b-5p expression is negatively correlated with the expression of some down-regulated hub genes, including *CD36* and *CXCL12* in patients with COAD (**D**). Conversely, hsa-miR-375 and hsa-miR-378a are significantly decreased in COAD tumors compared to the controls among the four down-regulated co-DEMs in the TCGA database (**E**). Among these miRNAs, only the hsa-miR-375 expression is negatively correlated with some up-regulated hub gene expression, including *CLDN1* and *INHBA,* in patients with COAD (**F**). *** *p*-value < 0.001.

Using GEO2R, we analyzed three datasets (GSE18392, GSE35982, and GSE41655) and identified a total of 240 up-regulated and 144 down-regulated DEMs. Reciprocal volcano maps for each dataset illustrate the distribution of significantly altered miRNAs (Figure 5A). Notably, cross-analysis revealed 8 co-DEMs consisting of up-regulated hsa-miR-135b (also known as has-miR-135b-5p), hsa-miR-183, hsa-miR-224, and hsa-miR-552 and down-regulated hsa-miR-30a, hsa-miR-375, hsa-miR-378a (also known as hsa-miR-378*), and hsa-miR-551b visualized in a Venn diagram (Figure 5B).

Consistent with our up-regulated DEMs, the TCGA dataset also showed significantly higher expression of all four DEMs in COAD tumors compared to the control (hsa-miR-135b: *p* < 1 × 10^−12^, hsa-miR-183: *p* < 1 × 10^−12^, hsa-miR-224: *p* = 2.7479 × 10^−9^, and hsa-miR-552: *p* = 1.6245 × 10^−12^) (Figure 5C). We examined the negative correlation between these key co-DEMs and hub gene expression to investigate the impact of up-regulated co-DEMs on down-regulated hub genes. Out of the 4 up-regulated co-DEMs, only has-mir-135b-5p exhibited a negative correlation, with the expression levels of *CD36* (r = −0.238, *p* = 3.38 × 10^−7^) and *CXCL12* (r = −0.408, *p* = 1.70 × 10^−19^), which were down-regulated hub genes (Figure 5D). By contrast, analysis of the TCGA dataset indicated that the key down-regulated co-DEMs were hsa-miR-375 (*p* < 1 × 10^−12^) and hsa-miR-378 (*p* = 1.61614 × 10^−5^), as these DEMs exhibited significant decreases in tumor compared to the control, while no significant difference was observed for hsa-miR-30a (*p* = 6.7714 × 10^−1^) and hsa-miR-551b (*p* = 3.981 × 10^−1^) between the groups (Figure 5E). To assess the influence of key down-regulated co-DEMs on up-regulated hub genes, we examined the negative correlation between these DEMs and their target genes. Among two key down-regulated DEMs, only hsa-miR-375 displayed a negative correlation, with the expression levels of *CLDN1* (r = −0.278, *p* = 1.97 × 10^−9^) and *INHBA* (r = −0.223, *p* = 1.86 × 10^−6^), which were up-regulated hub genes (Figure 5F).

### 3.5. Identification of DMRs Modulating the Expression of Hub Genes

**Figure 6:**
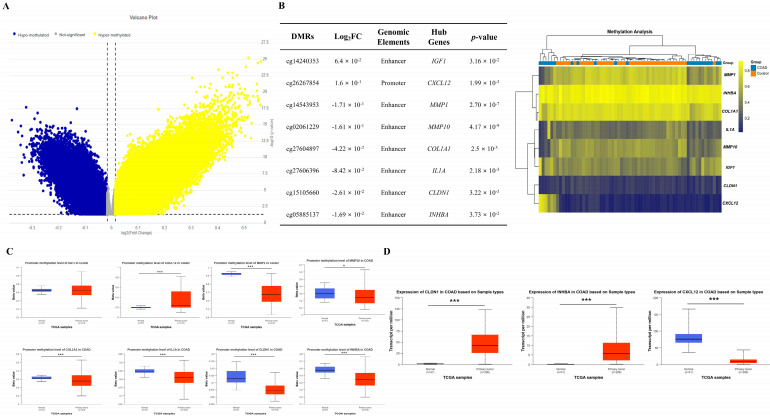
Expression levels of hub genes controlled by epigenetic regulation. Volcano map illustrating differently methylated region (DMR) distribution in GSE42752. In the volcano plots, yellow dots denote notably hyper-methylated DMRs identified with a fold change of ≥ 0.015 and a corrected *p*-value of <0.05. Conversely, blue dots represent hypo-methylated DMRs identified with a fold change ≤ −0.015 and a corrected *p*-value < 0.015 (**A**). Among the DMRs of various genes, the DMRs of hub genes with a negative correlation between methylation and expression are listed, and the methylation levels of these genes are depicted in a heatmap using color coding. Yellow signifies hyper-methylation, blue denotes hypo-methylation, and gray indicates no significant change (**B**). The promoter methylation levels of all hub genes listed in (**B**) exhibited significant differences between COAD tumor and control except for the *IGF1* in the TCGA database (**C**). Additionally, the expression levels of *CLDN1*, *INHBA*, and *CXCL12* are hub genes affected by both miRNAs and DNA methylation among hub genes. Analysis of the TCGA database revealed significantly increased expression levels of *CLDN1* and *INHBA*, while *CXCL12* exhibited significantly reduced expression levels (**D**). * *p*-value < 0.05, *** *p*-value < 0.001.

We conducted methylation profiling analysis on COAD tumor and normal tissue using GSE42752 dataset, revealing a total of 179,879 DMRs, comprising 78,343 hyper-methylated and 101,536 hypo-methylated CpG sites (Figure 6A). we analyzed the inverse correlation between methylation levels of DMRs and expression levels of hub genes to investigate the impact of methylation on hub gene expression. Among the hyper-methylated DMRs, cg14240353 and cg26267854 were identified in the enhancer and promoter regions of *IGF1* and *CXCL12*, respectively, which are down-regulated hub genes. Hypo-methylated DMRs, including cg14543953, cg02061229, cg27604897, cg27606396, cg15105660, and cg05885137, were located in the enhancer regions of *MMP1*, *MMP10*, *COL1A1*, *IL1A*, *CLDN1*, and *INHBA*, all of which are up-regulated hub genes (Figure 6B). In line with our findings of hyper-methylated DMRs in *CXCL12*, analysis of the TCGA dataset also revealed significant hyper-methylation of the *CXCL12* promoter (*p* = 1.62425 × 10^−12^) in COAD tumors compared to controls. However, there was no significant difference observed between the two groups in the case of *IGF1* promoter (*p* = 9.357 × 10^−1^). Conversely, analysis of the TCGA dataset revealed significant promoter hypo-methylation of *MMP1* (*p* = 1.62448 × 10^−12^), *MMP10* (*p* = 4.6901 × 10^−2^), *COL1A1* (*p* = 5.0124 × 10^−4^), *IL1A* (*p* = 1.41809 × 10^−12^), *CLDN1* (*p* = 3.33067 × 10^−16^), and *INHBA* (*p* = 1.62448 × 10^−12^), which is consistent with our findings from the DMR methylation analysis of the up-regulated hub genes (Figure 6C).

Remarkably, the expression levels of *INHBA*, *CLDN1*, and *CXCL12* among hub genes exhibited a pronounced negative correlation with both miRNAs targeting these genes and the DNA CpG site methylation regulating the epigenetic features of these genes. Additionally, the TCGA database indicated significantly higher expression of *CLDN1* and *INHBA*, while *CXCL12* displayed lower expression between COAD tumor and control samples, consistent with our analysis results of the DEGs, DEMs, and DMRs. (Figure 6D).

### 3.6. Expression Pattern Analysis of Hub Genes Using COAD scRNA-Seq Dataset

**Figure 7:**
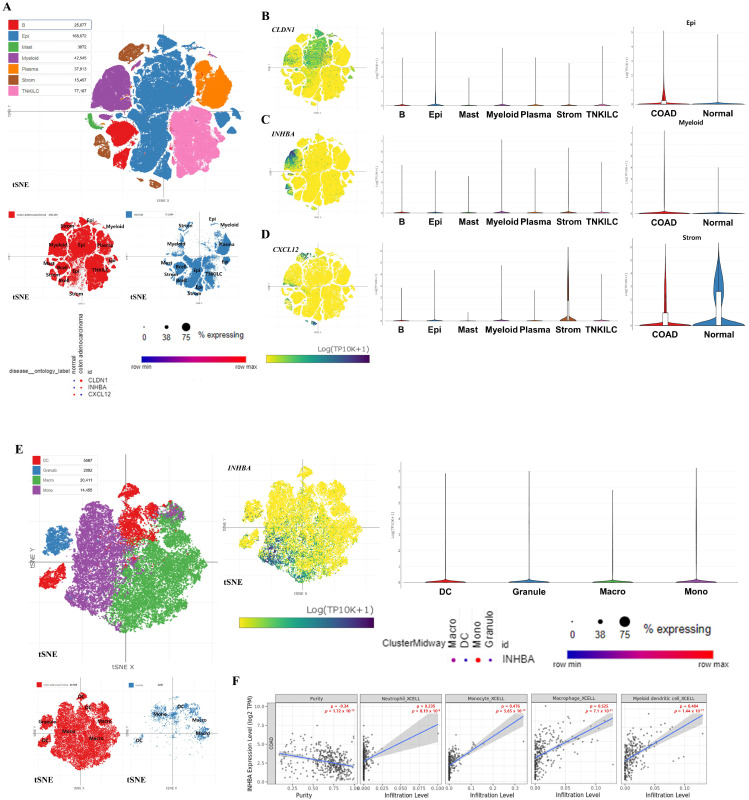
Expression patterns of the key hub genes and their correlation with immune cell infiltration. The entire cell types of COAD tumors and normal tissues and the expression levels of the three hub genes in all cell types are presented (**A**). Expression patterns of three hub genes and expression levels between COAD and control in the mainly expressed cell types (**B**–**D**). Expression pattern of *INHBA* in myeloid cells (**E**). All single-cell RNA sequencing data are depicted using the t-distributed Stochastic Neighbor Embedding (t-SNE) method. Immune infiltration patterns based on *INHBA* expression in myeloid cell types in COAD (**F**).

We performed scRNA-seq analysis on *CLDN1*, *INHBA*, and *CXC12*, regulated by both miRNAs and DNA methylation, in COAD tumor and normal colon tissue using GSE178341. Figure 7A illustrates the overall cell types of patients with COAD and control. The dot plot showed that the expression of *CLDN1*, *INHBA*, and *CXCL12* exhibited identical expression regulation with our previous analysis results in overall cell types (*CLDN1* and *INHBA* were up-regulated, and *CXCL12* was down-regulated, in COAD compared to the control. *CLDN1* is primarily expressed in epithelial cells, and *INHBA* is predominantly expressed in myeloid cells, with *CLDN1* and *INHBA* displaying increased expression levels in epithelial cells and myeloid cells in COAD compared to the control, respectively (Figure 7B,C). Conversely, *CXCL12* was mainly expressed in stromal cells and exhibited decreased expression levels in these cell types (Figure 7D). Among these three hub genes, only *INHBA* showed association with immune cell types, particularly being prominently expressed in monocytes among various myeloid cell types, such as dendritic cells (DCs), granulocytes, and macrophages (Figure 7E).

### 3.7. Correlation Analysis Between Immune Cell Infiltration and INHBA Expression Levels

To investigate the relevance between immune cell infiltration, particularly in cells expressing *INHBA*, and the level of *INHBA* expression, we calculated Spearman’s rho values on these elements in 458 COAD samples using the XCELL algorithm (Figure 7F). Consistent with the scRNA-seq results on *INHBA* expression levels in myeloid cells, monocytes (Spearman ρ = 0.476, *p* = 5.65 × 10^−17^) exhibited a significantly positive correlation between infiltration and *INHBA* expression levels in COAD. Furthermore, other myeloid cells, such as macrophages (Spearman ρ = 0.525, *p* = 7.10 × 10^−21^), granulocytes (especially neutrophils) (Spearman ρ = 0.235, *p* = 8.19 × 10^−5^), and DCs (Spearman ρ = 0.484, *p* = 1.44 × 10^−17^), also showed a notably positive correlation according to gene expression.

### 3.8. Tumor Progress Analysis Based on the Expression of Hub Genes

**Figure 8:**
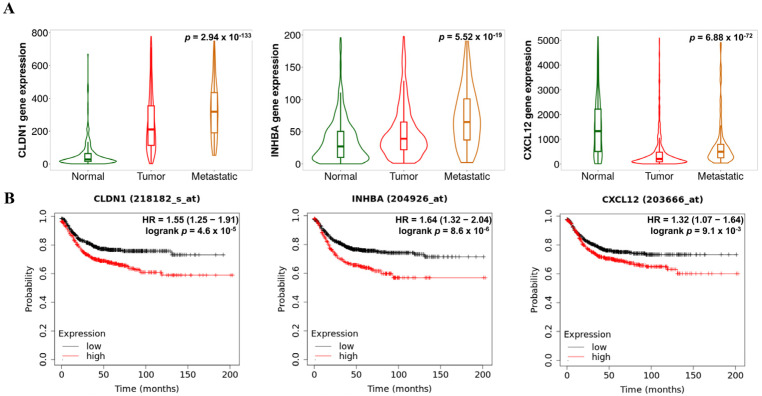
Prognostic patterns in COAD according to the expression levels of the key hub genes. Metastatic effects (**A**) and overall survival (OS) rates (**B**) based on *CLDN1*, *INHBA*, and *CXCL12* expression levels.

Figure 8A exhibited the relationship between expression of hub genes and tumor prognosis. Both *CLDN1* (*p* = 2.94 × 10^−133^) and *INHBA* (*p* = 5.52 × 10^−19^) expressions were significantly up-regulated in a tumor progression-dependent manner. However, in the case of *CXCL12*, the expression levels did not show down-regulation in a tumor progression-dependent manner (*p* = 6.88 × 10^−72^). Furthermore, Figure 8B demonstrated the relationship between hub gene expression and survival rates in patients with COAD. Consistent with the tumor metastatic analysis, high expression of *CLDN1* (hazard ratio (HR) > 1.3; *p* < 0.01) and *INHBA* (HR > 1.5; *p* < 0.001) was associated with poor prognosis, as indicated by a high HR. Additionally, high expression of *CXCL12* was also linked to poor survival rates (HR > 1.4; *p* < 0.001), despite our data suggesting that *CXCL12* was a down-regulated co-DEG.

## 4. Discussion

Understanding the underlying molecular mechanisms of COAD in terms of the TME would greatly benefit diagnosis, management, and prognosis evaluation. TME is a dynamic and complex network surrounding a tumor, consisting of various cell types, signaling molecules, and extracellular matrix components [29,30]. This environment plays a crucial role in tumor development, progression, and response to therapy. Two key regulatory mechanisms within the TME are miRNAs and DNA methylation, both of which significantly influence gene expression and cellular behavior [31,32]. The current study integrates extensive bioinformatics analysis to uncover novel biomarkers and molecular pathways associated with CRC, with a specific focus on COAD, which comprises the majority of CRC cases. By analyzing differential gene expression, miRNA regulation, and DNA methylation patterns, we identified key hub genes and pathways that may serve as critical therapeutic targets for personalized treatments.

Our analysis suggested the possible role of DEMs in regulating key hub genes. For example, hsa-miR-135b-5p showed negative correlation with *CD36* and *CXCL12*, both of which are down-regulated in COAD. hsa-miR-375 also displayed a negative correlation with up-regulated hub genes *CLDN1* and *INHBA*. These findings are consistent with previous reports demonstrating the involvement of miRNAs in tumorigenesis through post-transcriptional regulation, impacting processes like cell migration and invasion, and immune cell infiltration [33,34].

Epigenetic changes, specifically DNA methylation, emerged as another crucial regulatory mechanism influencing the expression of hub genes. We identified multiple DMRs in CpG islands, and enhancer and promoter regions of hub genes, correlating with altered gene expression. Notably, *CXCL12* exhibited hyper-methylation in their promoter regions, contributing to their down-regulation in COAD. Conversely, *MMP1*, *CLDN1*, *COL1A1*, and *INHBA* showed hypo-methylation in their enhancer regions, aligning with their up-regulation. These findings are consistent with the well-established role of DNA methylation in cancer, where hyper-methylation of tumor suppressor genes and hypo-methylation of oncogenes drive tumorigenesis [31].

We identified three hub genes—*INHBA*, *CLDN1*, and *CXCL12*—that showcase intricate regulatory processes involving both miRNA regulation and DNA methylation. These mechanisms are crucial for their expression levels and play a significant role in influencing tumor dynamics.

INHBA, part of the transforming growth factor β superfamily, is encoded in the nucleus of human cells, synthesized in the cytoplasm, and secreted through the membrane [35]. Recent research indicates that the *INHBA* is over-expressed in various cancers and is associated with cell proliferation and outcomes in lung [36], gastric [37], esophageal [38], and colorectal tumors [39]. A study on esophageal adenocarcinoma found higher *INHBA* expression in cancerous tissues compared to hyper-plastic esophageal tissues [38]. This suggests that *INHBA* over-expression may enhance cell proliferation and be influenced by promoter demethylation and histone acetylation in esophageal adenocarcinoma cell lines. Studies showed that overexpression of *INHBA* is positively correlated with poor prognosis in esophageal, prostate, and ovarian cancer [36,40,41]. In our study, through prognostic analysis, *INHBA* was recognized as correlating with poor prognosis in CRC patients. *INHBA* is often over-expressed in COAD tissues, correlating with increased tumor aggressiveness, higher metastatic potential, and poorer prognosis [42].

hsa-miR-375 functions as a tumor suppressor in many types of cancer. In colon cancer, hsa-miR-375 is often down-regulated, and its reduced expression is linked to poor patient prognosis [43,44]. Several studies have shown that while direct effects in CRC have not been definitively identified, hsa-miR-375 may target *INHBA* and potentially act to suppress its expression [45,46]. The reduction in hsa-miR-375 levels may diminish its suppressive impact on *INHBA*, potentially causing an upsurge in *INHBA* expression in CRC. Such a dysregulation could lead to more aggressive tumor characteristics, including increased cell proliferation and invasion, and a greater propensity for metastasis. The inverse relationship between *INHBA* and hsa-miR-375 in our study suggests that restoring the levels of hsa-miR-375 could potentially suppress *INHBA* activity, offering a therapeutic approach to inhibit tumor progression in colon cancer. This interaction highlights the importance of both *INHBA* and hsa-miR-375 as potential biomarkers and targets for personalized treatment strategies in CRC.

The CLDN family comprises at least 24 members, with their expression varying according to cell type [47]. CLDN1 is a crucial element of tight junctions and is vital in tumorigenesis [48]. CLDNs are responsible for regulating the differentiation, proliferation, and migration of epithelial cells [49]. Recent studies indicate that the expression of CLDN genes is frequently altered in cancers [50,51]. The role of CLDNs in cancer remains unclear; however, recent research suggests that the CLDN1-dependent pathway might play a role in suppressing CRC expression and is associated with tumor invasiveness and prognostic factors [49]. Studies have found that CLDN1 mRNA expression is elevated in CRC compared to normal colonic mucosa [52]. Moreover, CLDN1 has been linked to colon cancer tumorigenesis [53]. However, various studies have reported that claudin levels in cancer vary, with some showing increased expression [54,55] and others showing decreased expression levels [56,57]. In our study, *CLDN1* expression was significantly up-regulated in a tumor progression-dependent manner. Higher expression of CLDN1 was significantly associated with poor outcome.

Similarly to INHBA, our analysis showed an inverse relationship between *CLDN1* and hsa-miR-375. Over-expression of hsa-miR-375 down-regulates *CLDN1*, while knockdown of hsa-miR-375 up-regulates *CLDN1* in non-small cell lung cancer [58]. It is believed that a similar mechanism may operate in colon cancer. The down-regulation of hsa-miR-375, which is often observed in COAD, can lead to the up-regulation of *CLDN1*. This up-regulation can disrupt cell–cell adhesion due to changes in tight junction composition, facilitating enhanced cancer cell migration and invasion. The increased expression of *CLDN1* in CRC has been associated with poorer prognosis and may serve as a biomarker for invasive disease characteristics. Furthermore, the restoration of hsa-miR-375 levels might represent a therapeutic approach to mitigate these effects by repressing *CLDN1* expression, potentially inhibiting tumor progression and improving patient outcomes.

CXCL12 is an α-chemokine derived from stromal cells that encodes a family of interstitial anti-microbial genes. Previous study has indicated that the down-regulation of CXCL12 in CRC cell lines and primary tumor tissues may play a regulatory role in the initiation of CRC [59]. Experimentally, Wendt et al. reported that CXCL12 mRNA/protein is silenced by its promoter DNA hyper-methylation in primary colorectal tumor and cell lines [60]. The down-regulated CXCL12 is linked to tumor cells to resist anoikis, survive detachment, and circulate, since CXCL12 acts as a safeguard against metastasis by inducing anoikis [61]. These reports are consistent with our findings that *CXCL12* is hyper-methylated and down-regulated in COAD. Conversely, however, several studies have indicated that high levels of CXCL12 promote tumor growth, invasion, and poor prognosis in CRC [62]. CXCL12 and its receptor CXCR4 are crucial in the metastatic process of CRC [62]. The CXCL12/CXCR4 axis facilitates the migration and invasion of cancer cells to distant organs, particularly the liver and lungs, which are common sites of metastasis in CRC patients [63]. The CXCL12/CXCR4 interaction also promotes angiogenesis, the formation of new blood vessels, which is essential for tumor growth and providing nutrients to cancer cells [64]. CXCL12 contributes to the formation of a tumor-supportive micro-environment by recruiting immune cells, fibroblasts, and endothelial cells, which can aid in tumor growth and metastasis [62]. Due to its role in CRC progression and metastasis, CXCL12 and its receptor CXCR4 are considered potential therapeutic targets. Inhibiting the CXCL12/CXCR4 axis may provide a strategy to limit tumor growth and prevent metastasis in patients with CRC. Therefore, contradictory expression of CXCL12 might be related to early tumorigenesis and late metastasis in CRC.

hsa-miR-135b is an miRNA known for its roles in various cancers, acting either as an oncogene or a tumor suppressor depending on the context and tissue type. In colon cancer, hsa-miR-135b has been implicated in regulating several key genes involved in oncogenic pathways. hsa-miR-135b is known from several in vitro studies to directly down-regulate *CXCL12* [65]. In our research, it is also predicted that hsa-miR-135b negatively regulates *CXCL12* expression by binding to its mRNA and inhibiting its translation. This interaction can impact the CXCL12/CXCR4 signaling pathway, which is crucial for cancer cell migration and invasion. The dysregulation of hsa-miR-135b, leading to altered *CXCL12* expression, can significantly affect tumor behavior. Over-expression of hsa-miR-135b and subsequent down-regulation of *CXCL12* might reduce the chemotactic and angiogenic capabilities of cancer cells, potentially inhibiting metastasis.

*CLDN1*, *INHBA*, and *CXCL12* may serve as clinically relevant biomarkers in CRC, particularly as prognostic indicators linked to tumor progression, immune modulation, and metastatic potential. Over-expression of *CLDN1* has been associated with epithelial–mesenchymal transition, increased invasiveness, and metastatic behavior [66,67], as well as modulation of epithelial barrier permeability, suggesting a role in drug delivery and treatment efficacy—especially in ROS-based cancer therapies. Up-regulation of *INHBA* is linked to tumor proliferation, immune cell infiltration, and the development of an immunosuppressive micro-environment [68,69]. Its protein product, Activin A, promotes fibrosis, angiogenesis, and immune evasion, contributing to chemoresistance and poor prognosis in colorectal and gastric cancers [70]. CXCL12, through interaction with CXCR4, regulates tumor cell survival, invasion, and immune suppression [64,71]. High *CXCL12* expression is associated with lymph node metastasis and poor survival, and the CXCL12/CXCR4 axis facilitates the recruitment of regulatory T-cells and M2 macrophages that support tumor immune escape [64,71].

Despite the comprehensive nature of our multi-omics analysis, several limitations should be noted. Our findings are entirely based on publicly available datasets and computational algorithms, and do not provide direct biological or clinical validation. Without experimental evidence, the mechanistic and causal roles of these key hub genes in CRC pathogenesis cannot be fully established. Therefore, rigorous experimental validation is urgently required. Future studies must include in-vitro and in-vivo assays—such as knock-down, over-expression, epigenetic editing of key hub genes, and tumor modeling—to confirm their biological function and oncogenic potential. These experiments are essential not only to substantiate our findings, but also to evaluate their suitability as therapeutic targets. Moreover, prospective clinical investigations are necessary to assess their prognostic and predictive power across treatment modalities, including chemotherapy, immunotherapy, and epigenetic therapy. Further studies should also examine the regulatory interactions of miR-375 and miR-135b through functional miRNA assays. Ultimately, integrating these biomarkers into non-invasive diagnostic platforms—such as exosomal miRNA profiling or ctDNA methylation panels—could enable real-time disease monitoring and individualized treatment strategies. Such follow-up studies are not optional but represent a critical next step for the clinical translation of bioinformatics-driven discoveries in CRC.

Clinical Recommendations

- *CLDN1* and *INHBA* are consistently over-expressed in COAD and are associated with poor prognosis and tumor progression, suggesting their potential role as negative prognostic biomarkers.
- *CXCL12* is down-regulated and epigenetically silenced in COAD and may be in-volved in early tumor suppression, offering insight into immune micro-environment modulation.
- Epigenetic regulation (via miRNAs and DNA methylation) plays a critical role in gene dysregulation in COAD and may represent therapeutic targets or predictive markers.
- Integrated multi-omics analysis improves the identification of functionally relevant and clinically applicable biomarkers for personalized treatment strategies in COAD.

## 5. Conclusions

Our integrative multi-omics analysis identified *CLDN1*, *INHBA*, and *CXCL12* as key biomarkers in COAD, regulated by both miRNAs and DNA methylation. These genes showed significant associations with tumor progression, immune infiltration, and patient prognosis. Our findings provide insight into the molecular landscape of COAD and suggest that multi-layered biomarkers may guide the development of personalized treatment strategies. Further experimental and clinical validation is warranted to translate these insights into therapeutic applications.

## Data Availability

The data used in this study are available from the NCBI-GEO (https://www.ncbi.nlm.nih.gov/geo/, accessed on 15 January 2024), UALCAN (https://david.ncifcrf.gov, accessed on 5 February 2024), ENCORI/starBase (https://rnasysu.com/encori/, accessed on 19 February 2024), Single-Cell Portal database (https://singlecell.broadinstitute.org/single_cell, accessed on 12 February 2024), TIMER 2.0 (http://timer.cistrome.org/, accessed on 12 February 2024), Tumor, Normal, and Metastatic tissues plot.com database (https://tnmplot.com/, accessed on 19 February 2024), and KM plot database (https://kmplot.com/, accessed on 19 February 2024) databases, as well as, upon reasonable request, from the corresponding author.
